# Management of Knee Flexion Contracture in a Child With 3MC Syndrome Using Taylor Spatial Frame

**DOI:** 10.7759/cureus.17403

**Published:** 2021-08-24

**Authors:** Rayan A Alloqmani, Mohammed S Al-Zahrani, Gamal O Al-Tamimi, Emad A Bahmead, Mohammed H Al-Rumaih

**Affiliations:** 1 Orthopaedic Surgery, Prince Sultan Military Medical City, Riyadh, SAU; 2 Orthopaedic Surgery, Taibah University, Almadinah Almunawarah, SAU

**Keywords:** 3mc syndrome, flexion contracture, deformity, taylor spatial frame, autosomal recessive

## Abstract

3MC syndrome is a rare genetic disorder inherited through an autosomal recessive inheritance pattern caused by mutations in one of three genes: *COLEC11*, *COLEC10*, and *MASP1*. High-arched brows, ptosis, blepharophimosis, hypertelorism, cleft lip, cleft palate, developmental delay, hearing loss, abdominal wall defect, and urogenital and skeletal abnormalities are all characteristics. In previous reports, involvement of knee flexion contracture was not known to be one of the 3MC syndrome symptoms. The prevalence of 3MC syndrome is still unknown, and there have only been a few reports. We report the case of a four-year-old female with 3MC syndrome who was diagnosed with a confirmed mutation in the *COLEC11* gene. We describe a method for decreasing knee flexion contracture in a reported patient that makes use of the Taylor spatial frame (TSF). Accepted results were observed because the patient has full extension, which must be maintained by the brace. According to our findings, the TSF was the safest, most accurate, stable fixator, and most efficient solution for treating knee flexion contracture, resulting in high patient and family satisfaction.

## Introduction

3MC syndrome is a rare autosomal recessive disorder consisting of four other rare autosomal recessive disorders. The Mingarelli, Malpuech, Michels, and Carnevale syndromes were discovered to be distinct entities sharing some characteristics [1]. Mental retardation, intrauterine and postnatal growth restriction, hypertelorism, cleft palate, cleft lip, and urogenital anomalies are all characteristics of Malpuech syndrome [2]. Carnevale syndrome is characterized by downward slanted eyes, ptosis, blepharophimosis, strabismus, an extremely arched palate, a defect in the abdominal wall, a depressed nose bridge, hip dysplasia, and elbow contracture [3]. Mingarelli syndrome is similar to Carnevale syndrome in terms of clinical features, but it also includes flattening of the skull base, scoliosis, spina bifida, and radioulnar synostosis [4]. Michels syndrome is characterized by blepharoptosis, blepharophimosis, epicanthus inversus, an anterior chamber defect, and a short fifth finger [5]. In 3MC patients, an autosomal recessive inheritance pattern was observed. Two mutated genes, *MASP1* and *COLEC11*, are responsible for 3MC syndrome [6]. Both genes are involved in the complement lectin pathway, which is involved in innate immunity [6]. Additionally, *COLEC10* was discovered to be mutated in 3MC syndrome, resulting in craniofacial manifestations [7]. There are no previous reports discussing knee flexion contracture and its management in patients with 3MC syndrome. Contracture of the knee is one of the most disabling deformities. On the other hand, acute management of flexion knee contracture via soft tissue or bony procedures has the potential to result in catastrophic complications [8]. Gradual correction of knee flexion contracture using distraction histogenesis techniques and external fixation can prevent acute soft tissue injury and decrease the likelihood of recurrence [9].

## Case presentation

**Figure 1:**
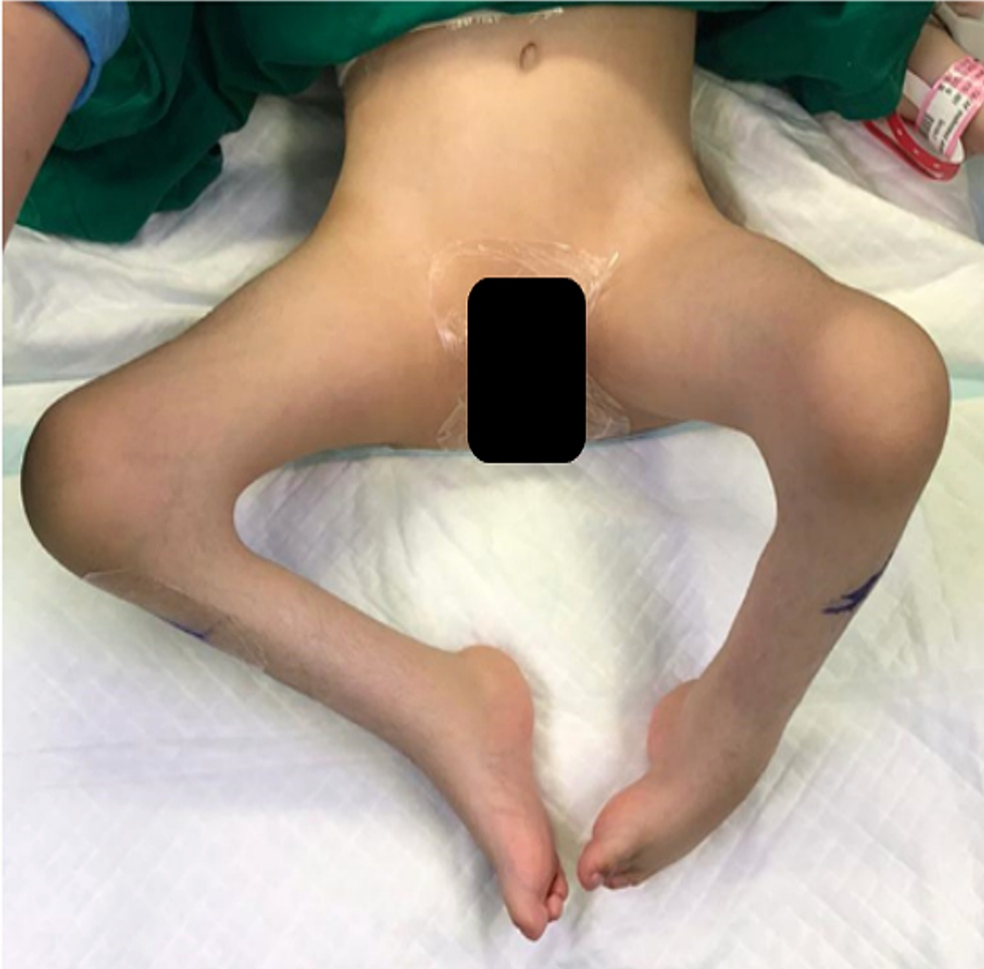
The preoperative image showed both knees with flexion contracture at 90 degrees.

A four-year old Saudi girl was referred for genetic analysis after birth due to dysmorphic features. Diagnosis of 3MC syndrome was obtained with *COLEC11* gene mutation. Family history was unremarkable. She was born prematurely at 34 weeks with a weight of 15 grams, a height of 42 cm, and a head circumference of 30 cm. Upon physical examination, her height and weight were 89 cm and 107 g, respectively. She is alert, and has good attention and concentration. She follows the simple command and has a speech level of lallation. All muscle groups are grossly graded as 4/5 on upper limbs and 4/5 on lower limbs. She has good head and trunk control. She can roll over, sit with fair balance, creep, and stand on both knees without an assistant. She is right-handed and has adequate hand function. She presented with facial dysmorphic features including blepharospasm, high-arched eyebrows, ptosis, hypertelorism, epicanthus inversus, cleft palate, and cleft lip. She also showed a hypoplastic of labia majora, bilateral knees fixed flexion contracture, and talipes equinovarus bilaterally. In addition, she also had limited right elbow extension about 10 degrees due to radial head subluxation in the right side posteriorly (Figure 1) .

**Figure 2:**
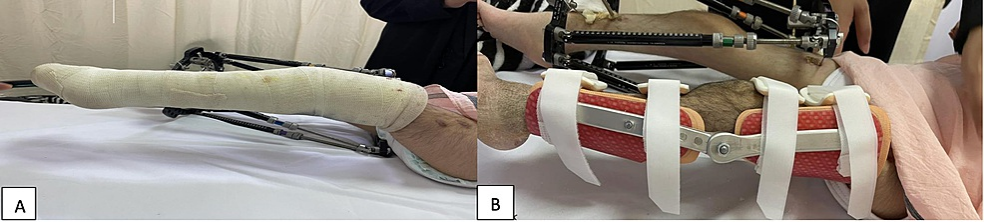
The left knee was maintained in full extension using an above knee cast (A) and a brace (B).

We started to address and manage both knees' fixed flexion contracture. Fixed flexion deformity of both knees was at 90 degrees. Gradual correction and straightening of both knees' flexion contracture by using Taylor spatial frame (TSF) was done. The TSF was applied with one ring above and one ring below the knee joint. The two rings were connected using six struts. Left-side TSF was applied for six weeks. Then, TSF was removed and the leg fixed with a complete above knee cast in full extension for six weeks (Figure 2).

**Figure 3:**
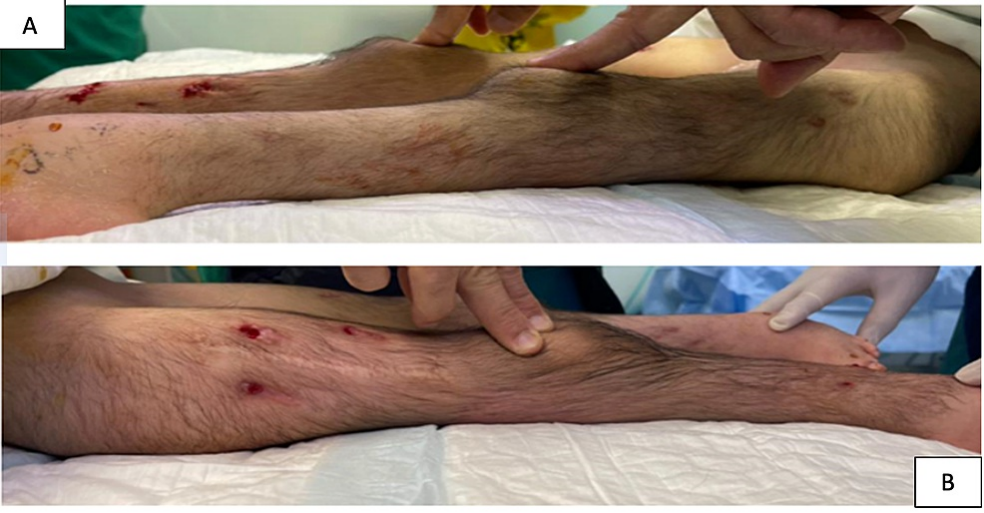
The postoperative image showed both knees in full extension (A, B).

Right-side TSF applied simultaneously when removed from the left side with the same duration. Then, the cast was applied for six weeks after removal from the right side. After removal of the cast, the patient’s knees were both in good alignment with full extension (Figure 3). 

## Discussion

We discovered a homozygous variant in the *COLEC11* gene in our reported patient. Additionally, in a heterozygous state, this variant was detected in both of the patient's parents. Our patient's 3MC syndrome was caused by a *COLEC11* gene mutation. seventeen patients with 3MC have been diagnosed with a mutated *COLEC11* gene [10]. Up to 2020, the literature has identified approximately 46 patients with 3MC syndrome from 34 families [10]. Twenty-six patients from 20 families were found to have an *MASP1* gene mutation, 17 individuals from 12 families were found to have a *COLEC11* gene mutation, and three individuals from two families were found to have a *COLEC10* gene mutation [6,8,11]. Individuals with the same mutation may exhibit distinct symptoms in 3MC syndrome [12]. The most noticeable symptoms in patients with 3MC syndrome are orbital hypertelorism, ptosis, blepharophimosis, epicanthus inversus, and highly arched eyebrows, as observed in our reported patient . Gardner et al. [13] reported one case of a patient with 3MC syndrome who is wheelchair-bound and has spastic contractures of the lower extremities without providing details about the patient's condition or treatment. There have been no other cases of 3MC syndrome affecting motor function reported to date. Our case demonstrates the possibility of flexion contracture deformity being involved in 3MC syndrome. As long as there are insufficient reports of 3MC patients, we must consider the possibility of additional contributing factors.

Knee flexion contracture is associated with significant physical disability. Avoid acute correction of knee flexion deformity to prevent serious complications [8]. Thus, progressive correction of deformity will help to minimize complications [9]. TSF is a hexapod external fixator that is integrated into a computer program for the purpose of correcting deformities. The ability of the TSF to correct six-axis deformity with greater precision and stability than other external fixators, in our opinion, makes it the preferred deformity correction device [14]. Numerous reports have demonstrated positive and accepted outcomes for knee flexion contracture correction using the TSF, confirming its accuracy, reliability, and safety in the management of deformities [14,15]. Vulcano et al. [9] provide evidence that gradual distraction with a circular external fixator is a safe and effective technique for treating knee flexion contracture. Maintaining the correction with a brace for a minimum of one to three months, depending on the severity of the contracture, is critical. In our reported patient, full extension of both knees was maintained for a total of three months using a cast and then a brace. 

In our reported patient, we observed a clinical improvement in preoperative and postoperative range of motion in both knees, as well as patient and family satisfaction. This is the first study to demonstrate the use of gradual distraction via TSF to correct knee flexion contracture in a patient with 3MC syndrome without soft tissue release or bony procedure. In our opinion, the TSF is the safest, most accurate, stable fixator, and most efficient solution, resulting in high patient and parent satisfaction. While the TSF technique restored full extension to both knees, the patient still has another deformity in both feet that requires further management. Our study encountered no complications, and no pin tract infection occurred, as was common in previous studies. The study's limitations include the scarcity of 3MC syndrome cases in the literature and the absence of any previous case of knee flexion contracture in 3MC syndrome.

## Conclusions

Knee flexion contracture was not previously described in the literature as a manifestation of 3MC syndrome, but it could be considered one of the representative symptoms. This appears to be the first report to describe knee flexion contracture in 3MC syndrome patients treated with TSF gradual distraction. We believe that using TSF is the safest, most accurate, and efficient method for achieving satisfactory results. More reports are required to define the clinical presentation in such patients and the best management strategy.
